# Effectiveness of simulation-based interprofessional education for medical and nursing students in South Korea: a pre-post survey

**DOI:** 10.1186/s12909-020-02395-9

**Published:** 2020-11-26

**Authors:** Jihye Yu, woosuck Lee, Miran Kim, Sangcheon Choi, Sungeun Lee, Soonsun Kim, Yunjung Jung, Dongwook Kwak, Hyunjoo Jung, Sukyung Lee, Yu-Jin Lee, Soo-Jin Hyun, Yun KANG, So Myeong Kim, Janghoon Lee

**Affiliations:** 1 Office of Medical Education, Ajou University School of Medicine, Suwon, South Korea; 2 College of Nursing, Taegu Science University, Daegu, South Korea; 3 Department of Obstetrics & Gynecology, Ajou University School of Medicine, Suwon, South Korea; 4 Department of Emergency Medicine, Ajou University School of Medicine, Suwon, South Korea; 5 Department of Gastroenterology, Ajou University School of Medicine, Suwon, South Korea; 6 Department of Pulmonary and Critical Care Medicine, Ajou University School of Medicine, Suwon, South Korea; 7 Department of Pediatrics, Ajou University School of Medicine, Suwon, South Korea; 8 Ajou Center for Clinical Excellence, Ajou University School of Medicine, Suwon, South Korea

**Keywords:** South Korea, Interprofessional education, Medical student, Nursing student

## Abstract

**Background:**

Effective collaboration and communication among health care team members are critical for providing safe medical care. Interprofessional education aims to instruct healthcare students how to learn with, from, and about healthcare professionals from different occupations to encourage effective collaboration to provide safe and high-quality patient care. The purpose of this study is to confirm the effectiveness of Interprofessional education by comparing students’ attitudes toward interprofessional learning before and after simulation-based interprofessional education, the perception of teamwork and collaboration between physicians and nurses, and the self-reported competency differences among students in interprofessional practice.

**Methods:**

The survey responses from 37 5th-year medical students and 38 4th-year nursing students who participated in an interprofessional education program were analyzed. The Attitude Towards Teamwork in Training Undergoing Designed Educational Simulation scale, the Jefferson Scale of Attitudes Toward Physician-Nurse Collaboration, and the Interprofessional Education Collaborative competency scale were used for this study. The demographic distribution of the study participants was obtained, and the perception differences before and after participation in interprofessional education between medical and nursing students were analyzed.

**Results:**

After interprofessional education, student awareness of interprofessional learning and self-competency in interprofessional practice improved. Total scores for the Jefferson Scale of Attitudes Toward Physician-Nurse Collaboration did not change significantly among medical students but increased significantly among nursing students. Additionally, there was no significant change in the perception of the role of other professions among either medical or nursing students.

**Conclusions:**

We observed an effect of interprofessional education on cultivating self-confidence and recognizing the importance of interprofessional collaboration between medical professions. It can be inferred that exposure to collaboration situations through Interprofessional education leads to a positive perception of interprofessional learning. However, even after their interprofessional education experience, existing perceptions of the role of other professional groups in the collaboration situation did not change, which shows the limitations of a one-time short-term program. This suggests that efforts should be made to ensure continuous exposure to social interaction experiences with other professions.

## Background

Effective collaboration and communication among health care team members are critical for providing safe patient care. Many health care accidents are associated with communication problems within the health care team and a lack of teamwork skills [1]. Interprofessional collaboration is important in that it increases job satisfaction of all health care team members including physicians, nurses and all allied health team members, positively affects patient satisfaction, and ultimately improves the quality of patient care [2, 3]. Implementation of interprofessional training within medical education is necessary for ensuring patient safety. Within the healthcare team, interprofessional education (IPE) should be initiated as an undergraduate course to cultivate effective communication and collaboration skills among future medical personnel.

IPE aims to instruct healthcare professionals how to learn with, from, and about one another to encourage effective collaboration for providing safe and high-quality patient care [4]. Thus, IPE can be implemented to improve students’ clinical performance so that effective communication, collaboration, and teamwork within the health care team can be achieved in the clinical setting [5]. As awareness of its importance grows, IPE is being increasingly implemented in healthcare-process education and studies have shown that through IPE, participating students developed a positive attitude toward interprofessional learning and that their collaboration skills improved [6].

Use of simulations in IPE is increasing [7] and, with developments in medical technology, high-fidelity simulation is increasingly used as a teaching-learning tool for health professionals [8]. Simulation is an effective tool for medical education [9] because it can function like a real patient care setting, and has been shown to improve students’ knowledge and communication skills [10, 11]. Simulation-based IPEs are drawing attention in the medical field because they provide students’ with opportunities to collaborate, communicate, make decisions, and practice skills among their team members in life-like situations; additionally, students can experience the consequences that arise from their decisions [12]. Furthermore, simulation education allows medical practice to be performed in a safe environment without the burden or pressures from the actual medical environment [13]. A study conducted on students participating in interprofessional simulation [14] showed that simulation-based IPE fosters communication skills between professions, enhances understanding of other occupations’ roles, enables collaboration, and improves self-confidence in collaborative situations, thus demonstrating its educational effectiveness.

Despite the growing awareness of the importance of Simulation-based IPE and related studies, there have been no cases in Korea where simulation-based IPE programs have been implemented and analyzed for medical and nursing students who will play key roles within healthcare teams. The purpose of this study is to analyze the educational effect of simulation-based IPE for medical students and nursing students in Korea. The specific research questions are as follows:

- What is the attitude of medical and nursing students to professional training, awareness of teamwork, and collaboration within the health care team, and competency for interprofessional collaborative practice?
- Are there pre- and post-training differences in students’ attitudes toward inter-professional training, awareness of teamwork and collaboration within the health care team, and competency for interprofessional collaborative practice?

## Methods

### Design

This study evaluated pre- and post-measures through quantitative surveys completed by medical and nursing students who participated in a high-fidelity simulation-based interprofessional program.

### Ethical considerations

This study was approved by the Institutional Review Board (IRB) of Ajou University Hospital (Ethics consent No. AJIRB-SBR-SUR-20-125).

### Setting

The simulation-based interprofessional program conducted from November 26–27, 2019 consisted of a total of three sessions: an adult simulation scenario with a patient complaining of chest pain (Module 1), a maternal scenario of a postpartum hemorrhage (Module 2), and a pediatric scenario in which a child presents with mild viral croup (Module 3). Module 1 aimed to collect information from patients complaining of chest pain to a team of emergency room doctors and nurses who performed the initial treatments. The Module 2 scenario was structured to test students’ ability to recognize changes in a patient with postpartum hemorrhage and their ability to engage in effective therapeutic communication with team members so that necessary medical procedures can be performed effectively. The Module 3 scenario aimed to evaluate the health care team’s ability to recognize symptoms in a child with febrile seizure symptoms due to viral croup and their ability to employ the necessary medical treatment skills, and to collaborate and communicate among the health care team. Each simulation module used in this study was determined by coordinating the medical and nursing faculty among the required graduation achievements of the university, and the simulation scenario was completed by adjusting the difficulty level through consultation. Although the scenario cases for each module are different, the task to be performed is the same, including diagnosis, treatment planning, and treatment action for patients through communication and collaboration between physicians and nurses. Therefore, the module was constructed so that students would have almost the same experience no matter which of the three modules they participated in.

### Procedures

A total of 87 students, including 43 medical students and 44 nursing students, participated in the simulation-based IPE program. Students were divided into 22 groups of 3–4 students per group. The 21 teams consisted of 2 medical and 2 nursing students each, and 1 team consisted of 1 medical and 2 nursing students. Two of the three modules were randomly assigned to each team. Therefore, each team experienced a total of two scenario modules. The format and contents of each simulation session are shown in Table 1. In the pre-briefing session, the instructor explained the aims and process of the program to the participating students. In addition, a simulation scenario template was provided to students so that they can check the scenario setting, learning outcome, description of scenario, and patient information. In the pre-scenario session, students set up a plan for actual simulation, such as analyzing cases and assigning roles based on a given scenario template. In the task training session, students practiced tasks necessary for actual performance such as checking manikin and equipment in the simulation room. In the simulation session, students intervened according to manikin actions based on programming data, and in this process, team members communicated and collaborated with each other. The students in the previous group who experienced the simulation module performed peer evaluation while watching what the students in the next group did. Through peer evaluation, students were able to reflect on their own performance, which serves as a debriefing. In addition, after the simulation session of each module was over, debriefing was conducted with the instructor on the performance of the group.

**Table 1:** Components of the simulation sessions

Session component	Time	Content
Pre-briefing	20 min	Introduction program objectives and operational processes, and obtain consent to record simulations
Pre-scenario activities	20 min	Situation analysis, coping plan, role sharing, and necessary task determination
Task training	20 min	Required task practice
Simulation	20 min	Scenario performance (different groups performed different modules), peer evaluation
Debriefing	20 min	Share thoughts on interprofessional experience

### Participants

A total of 87 students participated in this program, 43 medical and 44 nursing students. The data of participating students who did not respond to or responded insincerely to the survey to verify the effectiveness of this study were excluded. Thirty-seven fifth-year medical students and 38 fourth-year nursing students participated in the IPE program, and a total of 75 response variables were used for the analyses. Medical students in the 5th grade have competencies for essential treatments and clinical skills through major clinical clerkship, and have basic competencies for coping with emergency situations. In addition, the 4th grade nursing students have completed all clinical practice and have the basic knowledge and skills required in the medical situation at the hospital. In other words, it can be seen that the participants of this study are at the stage before entering the job and have the ability to evaluate their own capabilities. Missing responses or insincere responses were excluded. Students completed the questionnaire before and after IPE participation.

### Measures

In our study, the attitude toward interprofessional learning was measured using the Attitude Towards Teamwork in Training Undergoing Designed Educational Simulation (ATTITUDES) scale which was developed by Sigalet et al. [15]. This scale consists of 30 items organized into five sub-factors: IPE relevance (7 items, e.g., I want more opportunities to learn with other professionals.), simulation relevance (5 items, e.g., Simulation is a good tool for practicing team decision-making skills.), communication (8 items, e.g., Communication within the team is as important as technical skills.), situation awareness (4 items, e.g., Patient care is improved when all team members have a shared understanding about assessment and treatment.), and roles and responsibilities (6 items, e.g., Monitoring what each team member is doing is important for optimizing patient safety.). Each question was measured on a five-point scale from “strongly disagree” (1 point) to “strongly agree” (5 points). It can be seen that the higher the total score, the more positive students’ attitude toward interprofessional learning through simulation-based IPE. In this study, the pre and post-IPE questions show internal consistency (Cronbach α = 0.962 and 0.985, respectively).

To measure the perception of teamwork and collaboration between physicians and nurses, Jefferson Scale of Attitudes toward Physician-Nurse Collaboration (JSAPNC) developed by Hojat et al. [16] was used in this study. This scale consists of 15 items and 4 sub-factors: shared educational and collaborative relationships (7 items, e.g., Interprofessional relationships between physicians and nurses should be included in both professions’ educational programs.), caring as opposed to curing (3 items, e.g., Nurses are qualified to assess and respond to psychological aspects of patients’ needs.), nurse’s autonomy (3 items, e.g., Nurses should be accountable to patients for the nursing care they provide.), and physician’s authority (2 items, e.g., Doctors should be the dominant authority in all healthcare matters.). Each item was measured on a four-point scale from “strongly disagree” (1 point) to “strongly agree” (4 points). The higher the total score, the more positively students perceive teamwork and collaboration between physicians and nurses. The Cronbach α for inter-consistency before and after IPE was 0.911 and 0.935, respectively.

The Interprofessional Education Collaborative (IPEC) Competency self-assessment tool developed by Lockeman [17] was used to measure students’ competency in interprofessional practice. This scale consists of two sub-factors: interprofessional interaction (7 items, e.g., I can use strategies that will improve the effectiveness of interprofessional teamwork and team-based care.) and interprofessional value (9 items, e.g., I can embrace the diversity that characterizes patients and the healthcare team.). Each item was measured on a 5-point Likert scale from “strongly disagree” (1 point) to “strongly agree” (5 points). It can be seen that the higher the total score, the more students perceived as more competent in interprofessional practice. The Cronbach α for competency before and after IPE was 0.957 and 0.980, respectively.

### Analyses

Descriptive statistical analyses were conducted to identify the demographic distribution of participants in this study, and t-tests were conducted to explore response differences between the professional groups of medical and nursing students. Additionally, paired t-tests were conducted using SPSS version 25.0 (IBM, Armonk, NY, USA) to identify changes in medical and nursing students’ scores before and after IPE participation.

## Results

### Demographics

Participant characteristics are presented in Table 2. There were 1.7 times more male students than female students among the medical-school respondents, while there were 4 times more female students than male students among the nursing-school respondents. Medical students had no prior experience in simulation training, but nursing students had undergone previous simulation training. None of the participating students had any educational experience with other professions.

**Table 2:** Participant demographic data (*N* = 75)

	Medical	Nursing
Gender		
Male	24	7
Female	13	31
Previous experience with simulation sessions		
Yes	0	38
No	37	0
Previous experience with interprofessional education		
Yes	0	0
No	37	38

ATTITUDES, JSAPNC, and IPEC Competency scores according to profession are presented in Table 3. The differences between ATTITUDES, JSAPNC, and IPEC Competency were compared and analyzed for both student groups before and after IPE. Nursing students scored higher than medical students in ATTITUDES and IPEC Competency before and after IPE. The two groups did not differ significantly in JSAPNC scores before or after IPE.

**Table 3:** ATTITUDES, JSAPNC, and IPEC Competency scores according to profession

	ATTITUDES	JSAPNC	IPEC Competency
Pre-IPE			
Medical	4.18 (0.48)	3.25 (0.38)	3.97 (0.66)
Nursing	4.50 (0.39)	3.36 (0.42)	4.48 (0.49)
t	3.15^**^	1.14	3.77^***^
Post-IPE			
Medical	4.45 (0.73)	3.36 (0.60)	4.36 (0.78)
Nursing	4.77 (0.29)	3.52 (0.35)	4.78 (0.33)
t	2.55^*^	1.44	3.03^**^

Range of score: ATTITUDES (1–5), JSAPNC (1–4), IPEC Competency (1–5)

^*^p < 0.05, ^**^p < 0.01, ^***^p < 0.001

**Fig. 1:**
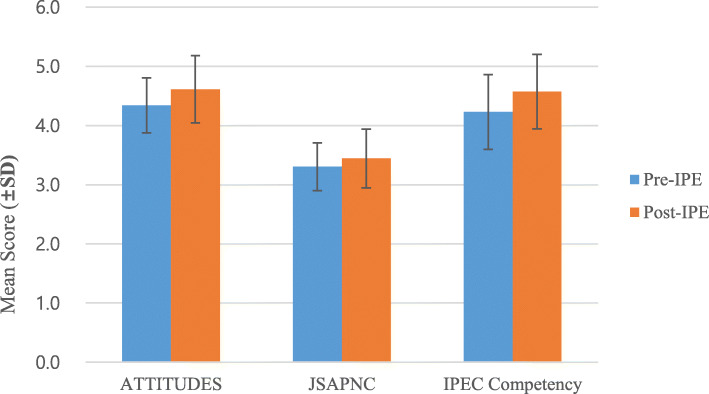
Comparison of ATTITUDES, JSAPNC, and IPEC Competency scores before and after simulation-based interprofessional education

The results of comparative analyses of the differences between the ATTITUDES, JSAPNC, and IPEC Competency scores before and after IPE experience are presented in Fig. 1.

All students participating in interprofessional education showed significant improvement in ATTITUDES, JSAPNC, and IPEC competency scores. Before the interprofessional education, the average scores of each scale were ATTITUDES (M = 4.34, SD = 0.47), JSAPNC (M = 3.30, SD = 0.40), and IPEC Competency (M = 4.23, SD = 0.63). After the program experience, the score of each scale was found to increase significantly as follows: ATTITUDES (M = 4.61, SD = 0.57, t = 4.63^***^), JSAPNC (M = 3.44, SD = 0.50, t = 2.87^**^), and IPEC Competency (M = 4.57, SD = 0.63, t = 5.25^***^).

Table 4 shows the ATTITUDES, JSAPNC, and IPEC Competency scale scores for medical and nursing students before and after participating in IPE programs. The overall score for nursing students was higher than that for medical students, but the score for physician’s authority, one of the sub-factors of the JSAPNC scale was higher among medical students (pre: M = 2.80 vs. M = 2.57, post: M = 2.92 vs. M = 2.72).

**Table 4:** Comparison of pre- and post-simulation scores for interprofessional education between medical and nursing students

Scale			Medical (***n*** = 37)		Nursing (***n*** = 38)	
			M (SD)	t	M (SD)	t
ATTITUDES	Relevance of IPE	Pre	4.10 (0.53)	2.35^*^	4.39 (0.47)	5.33^***^
		Post	4.35 (0.76)		4.75 (0.31)	
	Relevance of Simulation	Pre	4.16 (0.60)	2.61^*^	4.43 (0.49)	4.68^***^
		Post	4.46 (0.76)		4.81 (0.33)	
	Communication	Pre	4.21 (0.54)	2.09^*^	4.61 (0.38)	4.34^***^
		Post	4.46 (0.75)		4.85 (0.29)	
	Situation awareness	Pre	4.15 (0.49)	2.36^*^	4.45 (0.49)	3.51^**^
		Post	4.45 (0.78)		4.67 (0.41)	
	Roles and responsibility	Pre	4.27 (0.57)	1.90	4.61 (0.45)	3.24^**^
		Post	4.53 (0.76)		4.80 (0.33)	
	Total	Pre	4.18 (0.48)	2.50^*^	4.50 (0.39)	5.32^***^
		Post	4.45 (0.73)		4.77 (0.29)	
JSAPNC	Shared educational and collaborative relationships	Pre	3.29 (0.49)	2.36^*^	3.59 (0.40)	3.64^**^
		Post	3.51 (0.59)		3.77 (0.32)	
	Caring as opposed to curing	Pre	3.37 (0.47)	0.68	3.60 (0.49)	2.82^**^
		Post	3.43 (0.63)		3.79 (0.37)	
	Nurse’s autonomy	Pre	3.55 (0.47)	0.30	3.68 (0.44)	2.37^*^
		Post	3.58 (0.60)		3.81 (0.37)	
	Physician’s authority	Pre	2.80 (0.65)	0.81	2.57 (0.86)	1.18
		Post	2.92 (0.99)		2.72 (0.83)	
	Total	Pre	3.25 (0.38)	1.36	3.36 (0.42)	3.02^**^
		Post	3.36 (0.60)		3.52 (0.35)	
IPEC Competency	Interprofessional interaction	Pre	3.85 (0.78)	3.52^**^	4.38 (0.60)	4.31^***^
		Post	4.31 (0.83)		4.71 (0.41)	
	Interprofessional value	Pre	4.10 (0.62	2.93^**^	4.58 (0.46)	3.82^***^
		Post	4.41 (0.76)		4.85 (0.30)	
	Total	Pre	3.97 (0.66)	3.44^**^	4.48 (0.49)	4.34^***^
		Post	4.10 (0.53)		4.39 (0.47)	

^*^p < 0.05, ^**^p < 0.01, ^***^p < 0.001

The total scores from ATTITUDES (t = 2.50, *p* < 0.05 vs. t = 5.32, *p* < 0.001) and IPEC Competency (t = 3.44, *p* < 0.01 vs. t = 4.34, *p* < 0.00) increased significantly after IPE in both medical and nursing students. The total score for JSAPNC showed no significant change among medical students, but a significant increase among nursing students (t = 3.02, *p* < 0.01). Regarding changes in scores before and after IPE by JSAPNC sub-factors, in “shared educational and collaborative relationships,” which both measure the perception of collaboration between physicians and nurses, the scores from both medical students and nursing students increased (t = 2.36, *p* < 0.05). vs. t = 3.64, *p* < 0.01). For medical students, however, there was no change in scores for caring as opposed to curing, nurse’s autonomy, or physician’s authority, which measures the identity of each profession group. Also, there was no significant change in the score for physician’s authority among nursing students.

## Discussion

We found that nursing students were more positively aware of interprofessional learning and competency in interprofessional practice than medical students. Prior studies that compared the impact of IPE experience on the perception of interprofessional learning between the different professional groups have yielded mixed results. A study that compared attitudes to interprofessional learning before and after IPE [15] for different professional groups showed that medical students had more positive attitudes toward IPE than nursing students, which is a contrary finding to this study. This means that certain profession, nurses or physicians, do not always have a more positive attitude toward interprofessional learning than others. What direct or indirect experiences they had in patient care settings in previous clinical practice may be more important factors for attitude toward interprofessional collaboration and interprofessional learning [18].

Prior to this study, neither the nursing- nor medical-student participants had IPE experience, but medical students had 1 year of clinical experience practice, while nursing students, who were at the end of their fourth year, had almost 2 years of clinical experience practice and the nursing students had previously undertaken simulation in clerkship. Clinical experience practice provides positive and negative role modeling observations and collaboration among doctors, nurses, and other relevant allied health practitioners [19], and extensive exposure to situations that require collaboration with other professional groups may affect students’ perceptions of interprofessional learning [20]. It can be understood in the same context that the more experienced clinical practice, the higher the self-competency in interprofesssional practice. Therefore, one interpretation of our results is that nursing students with more clinical experience practice responded more positively toward interprofessional learning and self-competency in interprofessional situations than medical students. After simulation-based IPE, the attitude toward interprofessional learning for all participating professions improved. These results are consistent with a prior study [21] in which a simulation-based IPE program had a positive effect on the attitudes of medical and nursing students toward IPE. Additionally, after simulation-based IPE, the perception of all students who participated in teamwork and collaborations between physicians and nurses had improved.

However, sub-analyses of only medical students and only nursing students revealed no change in medical students’ perception about nurse autonomy nor nursing students’ perception about physician authority according to factors measuring perceptions of professional identity. In other words, we observed no change in students’ perception of the professional identities of the different profession groups, which is in contrast with a previous study [22, 23] that found changes in stereotypes of other professions after IPE. We infer that this is due to the cultural contexts of the rigid Korean medical organizations. Two days of short-term IPE is unlikely to change students’ perceptions of the roles of other professions compared to what they learned through their clinical training in the hospital. The point of focus between physicians and nurses is different in the clinical settings, the physicians are in charge of a directive role that makes the overall decision-making, and the nurse takes on a subservient role that focuses on patient care [24]. Our findings suggest that existing stereotypes about specific professions are challenges to be overcome in interprofessional learning. Beyond short-term special programs, it is necessary to include interprofessional collaborative training in clinical practice so that students can fully understand the role of other professions through the process of experiencing, observing, interacting, and reflecting positive modeling [25, 26].

After simulation-based IPE, self-reported competency improved for both medical and nursing students. Through IPE, students improved their ability to communicate and solve problems collaboratively among their health care team, and to understand team members and perform patient-centered care more effectively. A prior study [27, 28] found that interprofessional simulation improved self-competency in communication, collaboration, and situation management among team members in clinical settings. Competency development is a key component of clinical training [29]. Continuous provision of IPE improves competency, which helps postgraduates perform proficiently in their field of patient care. Patient care in clinical contexts always requires effective teamwork and communication skills among the health care team. However, because the current university education system is centered on majors, the necessary qualities for collaboration are insufficiently cultivated. IPE could be a good alternative approach to fill this learning gap.

This study is meaningful in that it is an empirical study that identified the educational effects of simulation-based IPEs in Korea, where IPE education has not yet been enacted. This study evaluated the educational effects of IPE, but this study has limitations in that it is a study on a single and short IPE. In order to analyze the effects of IPE in more detail, it is necessary to conduct IPE periodically and then perform analysis based on accumulated data. In addition, in analyzing the effects of the IPE program, there are limitations in that there may be various factors that can affect the study results in addition to the factors considered in this study. It is necessary to conduct a follow-up study that considers more various related variables such as student achievement. This study also has limitations in that it used an outdated scale that was developed to investigate the perception of teamwork and collaboration between physicians and nurses. When using it in future research, it is necessary to consider using it after going through an appropriate revision for the item according to the current situation. It also has an important limitation, in that it did not evaluate how long the effects of IPE will last. A reliable estimate of the effect duration is important for setting the cycle of education. It would then be necessary to conduct a study among postgraduates who participated in IPE as students to periodically evaluate the persistence of the educational effects of IPE. Additionally, in the long-term, when students embark on real clinical work after graduation, it is important to conduct research to evaluate whether healthcare professionals who had IPE experience during their training show better clinical performance through effective teamwork and communication.

## Conclusions

This study was conducted to assess attitudes toward interprofessional learning, perception of teamwork and collaboration between doctors and nurses, and self-reported competency of students in interprofessional practice, and to compare the differences before and after simulation-based IPE. Through IPE, students’ attitude toward interprofessional leaning and self-competency in interprofessional practice were improved. The perception of teamwork and collaboration between physicians and nurses showed no significant change among medical students but increased significantly among nursing students. Additionally, there was no significant change in the perception of the role of other professions among either medical or nursing students.

In this study, we found evidence for a positive educational effect of IPE in terms of participants recognizing the necessity of IPE and improving students’ confidence in their inter-professional collaboration abilities. However, the fact that the existing perception of the role of other professions did not change after IPE shows the limitations of a one-time short-term program. Efforts should be made to include programs within the clinical-practice curriculum that provide social interactions with other profession groups in clinical situations so that students can be continuously exposed to these experiences.

## Data Availability

The dataset used during the current study is available from the corresponding author upon reasonable request.

## Abbreviations

IPE: Interprofessional Education
ATTITUDES: Attitude Towards Teamwork in Training Undergoing Designed Educational Simulation
JSAPNC: Jefferson Scale of Attitudes toward Physician-Nurse Collaboration
IPEC: Interprofessional Education Collaborative
